# Molecular Detection of Hematozoa Infections in Tundra Swans Relative to Migration Patterns and Ecological Conditions at Breeding Grounds

**DOI:** 10.1371/journal.pone.0045789

**Published:** 2012-09-25

**Authors:** Andrew M. Ramey, Craig R. Ely, Joel A. Schmutz, John M. Pearce, Darryl J. Heard

**Affiliations:** 1 U.S. Geological Survey, Alaska Science Center, Anchorage, Alaska, United States of America; 2 College of Veterinary Medicine, University of Florida, Gainesville, Florida, United States of America; Institut Jacques Monod, France

## Abstract

Tundra swans (*Cygnus columbianus*) are broadly distributed in North America, use a wide variety of habitats, and exhibit diverse migration strategies. We investigated patterns of hematozoa infection in three populations of tundra swans that breed in Alaska using satellite tracking to infer host movement and molecular techniques to assess the prevalence and genetic diversity of parasites. We evaluated whether migratory patterns and environmental conditions at breeding areas explain the prevalence of blood parasites in migratory birds by contrasting the fit of competing models formulated in an occupancy modeling framework and calculating the detection probability of the top model using Akaike Information Criterion (AIC). We described genetic diversity of blood parasites in each population of swans by calculating the number of unique parasite haplotypes observed. Blood parasite infection was significantly different between populations of Alaska tundra swans, with the highest estimated prevalence occurring among birds occupying breeding areas with lower mean daily wind speeds and higher daily summer temperatures. Models including covariates of wind speed and temperature during summer months at breeding grounds better predicted hematozoa prevalence than those that included annual migration distance or duration. Genetic diversity of blood parasites in populations of tundra swans appeared to be relative to hematozoa prevalence. Our results suggest ecological conditions at breeding grounds may explain differences of hematozoa infection among populations of tundra swans that breed in Alaska.

## Introduction

In North America, hematozoa infection in wild birds varies across the landscape and among taxa [1]. Past studies have generally found low prevalence of blood parasites in taxa sampled at northern arctic and sub-arctic tundra landscapes [1]–[4] in contrast to higher rates of infection detected in birds sampled throughout forested habitats of Alaska and northern Canada [2], [5]–[6]. Low apparent prevalence of hematozoa in wild birds using tundra habitats may be due to lack of tree cover and concomitant high wind speeds that limit the availability of hosts to suitable vectors [2], or lower daily temperatures that inhibit hematozoa development [7]–[8]. Exposure of birds to blood parasites at wintering areas and/or en route to and from breeding grounds could be another important factor. Indeed, prevalence and diversity of hematozoa infections have been correlated with annual migration distance in waterfowl [9] and other avian taxa [10]. Therefore, ecological conditions at breeding grounds and migration patterns of birds may explain disparate rates of hematozoa infection, even within host species [7].

North American tundra swans (*Cygnus columbianus*) are divided into populations based on seasonal distribution and migratory patterns. In Alaska, birds that breed along the Arctic Coastal Plain migrate eastward during autumn and winter in the Atlantic Flyway [11]–[14], whereas birds that breed in western Alaska migrate down the Pacific Flyway [15]–[16]. A small and mostly non-migratory population resides on the Lower Alaska Peninsula [17]. Therefore, differences in breeding habitats and migratory patterns of North American tundra swans could contribute to variation in prevalence and genetic diversity of blood parasites.

In this study, we evaluated the relationships between migratory patterns, environmental conditions at breeding areas, and hematozoa prevalence in three populations of tundra swans. We also describe genetic diversity of hematozoa infecting swans from these three populations. We predicted tundra swans that breed and molt at high latitude tundra landscapes, where environmental conditions are likely less favorable for hematozoa development and transmission, would have a lower incidence of hematozoa infection than those using habitats adjacent to or within forest during summer [1]–[2]. Additionally, we predicted populations of tundra swans that migrate long distances to lower latitude wintering areas would have higher rates of infection and greater genetic diversity of blood parasites than those that are less migratory and remain in Alaska throughout the year [9]. We evaluated predictions about hematozoa prevalence by contrasting the fit of competing models formulated in an occupancy modeling framework [18], which allows estimation of prevalence while accounting for imperfect detection. Occasional failure of molecular techniques to detect hematozoa infections is a limitation of polymerase chain reaction (PCR) based diagnostic tests [19]–[20] which may occur for a variety of reasons (see Materials and methods). We assessed genetic diversity of blood parasites among populations by calculating the number of unique parasite haplotypes observed. Results of this study provide an assessment of evidence for differential parasite infection among three populations of tundra swans sampled in Alaska. Furthermore, results provide an empirical evaluation of conclusions from previous investigations pertaining to possible relationships between hematozoa prevalence and migration distance [9] and habitat characteristics [1]–[2] while controlling for potential species effects.

## Materials and Methods

### Ethics Statement

All procedures involving animals were approved by the U. S. Fish and Wildlife Service Region 7 and U. S. Geological Survey Alaska Science Center Animal Care and Use Committees. Animals were instrumented with satellite transmitters under the Department of Interior, U. S. Fish and Wildlife Service permit MB789758-5 (2008) and the State of Alaska, Department of Fish and Game scientific permit 2008-029. Blood samples were collected from tundra swans under the Department of the Interior, U. S Geological Survey Federal Bird Banding Permit #20022.

### Identifying Migratory Patterns of Alaska Tundra Swans

**Figure 1:**
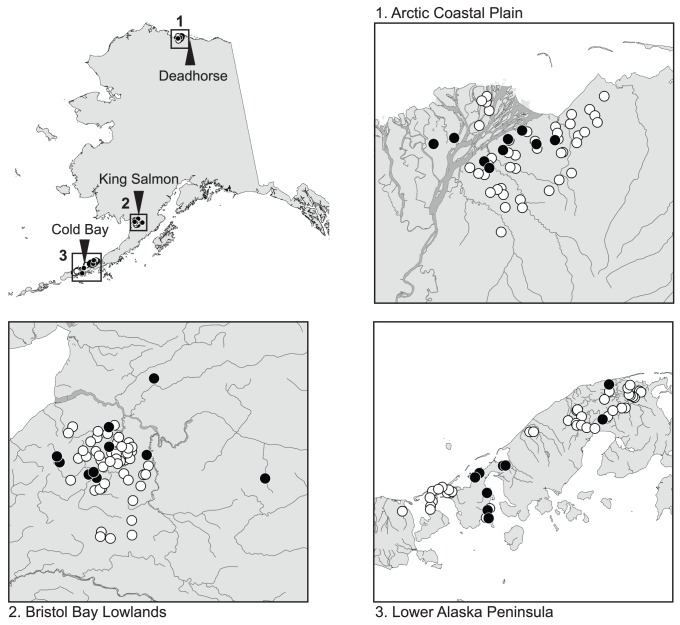
Capture locations of tundra swans implanted with satellite transmitters in July and August of 2008 (black circles) and bled in 2010 (white circles). The closest weather station to each of the three capture locations is indicated by the point of a black triangle.

Tundra swans were captured and marked with satellite transmitters (PTTs – Platform Transmitting Terminals) from mid-July through mid-August of 2008 at three molting locations in Alaska: the Arctic Coastal Plain (n = 10), the Bristol Bay Lowlands (n = 10) and the Lower Alaska Peninsula (n = 10; Figure 1). It should be noted that birds that use the Arctic Coastal Plain are considered to belong to the Eastern population and that those occurring at the Bristol Bay Lowlands and the Lower Alaska Peninsula are generally considered to be part of the Western population; however, swans are referenced by breeding/molting location in Alaska throughout this manuscript. The age of each bird (second-year or after-second-year) was assessed by plumage and sex was determined through cloacal examination. PTTs manufactured by Microwave Telemetry (Columbia, MD) were surgically implanted in second-year (12–14 months) or after-second-year (≥24 months) tundra swans. A single instrumented swan from each of the Arctic Coastal Plain and Bristol Bay Lowland populations was identified as male. All other individuals carrying transmitters were female. PTTs were programmed to transmit for 5 hours every 18 to 80 hours, depending upon season. The Argos Data Collection and Location System [21] was used to obtain information on latitude and longitude, date, time, and quality of locations of swans instrumented with PTTs. Unlikely locations were filtered based on rate and angle of movement [22], and the highest quality location was used to represent the daily position of swans.

### Detecting Hematozoa Infection

Whole blood samples were collected from molting second-year and after-second-year tundra swans at locations along the Arctic Coastal Plain (n = 100), the Bristol Bay Lowlands (n = 104), and the Lower Alaska Peninsula (n = 104) in July and August of 2010. Blood samples were collected from the jugular vein of each bird and preserved in Longmire buffer solution. DNA was extracted from all samples using the DNeasy Blood and Tissue Kit (Qiagen, Valencia, CA) according to the manufacturer’s protocol. Extracted DNA was screened for presence of hematozoa using a nested PCR as described by Hellgren et al. [19]. A minimum of one negative control every 24 wells was incorporated into each PCR reaction. Reactions were conducted in eight well strip tubes with individual caps which remained closed except while loading template and reagents to prevent cross contamination. Products were run out on 0.8% agarose gels. All samples were screened for hematozoa by nested PCR twice or until samples were identified as positive for both *Leucocytozoon* and *Haemoproteus*/*Parahaemoproteus/Plasmodium* parasites. Infections identified as *Haemoproteus/Parahaemoproteus/Plasmodium* were assessed through a single test and therefore were only distinguished by parasite genera through subsequent genetic sequencing and phylogenetic analysis.

The target fragment of 479 base pairs (bp) of parasite cytchrome *b* (cyt *b*) mitochondrial DNA (mtDNA) was sequenced for all samples detected as positive for hematozoa infection to provide information on haplotype diversity and to prevent misidentification resulting from co-amplification [20]. PCR products were treated with ExoSap-IT (USB Inc., Cleveland, OH) without additional purification prior to sequencing. Cycle sequencing was performed with identical primers used for PCR along with BigDye Terminator version 3.1 mix (Applied Biosystems, Foster City, CA) and analyzed on an Applied Biosystems 3730xl automated DNA sequencer (Applied Biosystems, Foster City, CA). Sequence data from *Leucocytozoon*, *Haemoproteus*/*Parahaemoproteus* and *Plasmodium* parasites was cleaned and edited using Sequencher version 4.7 (Gene Codes Corp., Ann Arbor, MI).

Additionally, a positive control PCR was performed that targeted a 470 bp fragment of the mtDNA cytochrome oxidase I gene of each sample to confirm competency of extractions. Reactions were conducted using 2 µl of template DNA, primers specifically designed for this study (TUSW COI F: GAT CGG GGG ATT TGG TAA CT and TUSW COI R: ACA GGA TTG GGT CTC CTC CT), and the same proportion of reagents as previously reported for the nested PCR [19]. Thermalcycling conditions followed Kerr et al. [23].

### Estimating Prevalence Using Model Selection and Occupancy Modeling

Samples were considered positive for hematozoa infection in analyses only if the 479 bp mtDNA cyt *b* product was visualized on a 0.8% agarose gel and confirmed by ≥90% max identity score of the double stranded target sequence as *Leucocytozoon*, *Haemoproteus*, *Parahaemoproteus*, or *Plasmodium* parasites using the nucleotide BLAST function available through the National Center for Biotechnology Information (NCBI). However, molecular techniques described above may have failed to detect hematozoa that were present in any given blood sample for a variety of reasons including: strong infection by one parasite masking weak infection by another [20], PCR inhibition, or degraded/low quantity template mtDNA. Thus, imperfect detection was accounted for and prevalence more accurately estimated using an occupancy modeling approach [18].

A central assumption of occupancy modeling is ‘demographic closure’ among two or more independent assessments of presence (e.g., if hematozoa are present in a blood sample, then they remain throughout all detection attempts). The basic premise of occupancy modeling in the context of this study is that, if hematozoa are detected in a sample in one assessment but not in a subsequent or previous assessment, then that constitutes a detection failure. Thus, occupancy modeling allows for estimation of the detection rate across the population of samples.

For this particular application, we did not expect there to be variation in the probability of detection among attempts to detect hematozoa within a given blood sample nor among samples because of controlled and consistent laboratory methods. However, we did expect there to be variation among groups of swans in the prevalence of hematozoa because of ecological differences among populations. Thus, we explicitly evaluated whether population affiliation, mean daily temperature at breeding areas, mean daily wind speed at breeding areas, annual migration duration by population, and annual migration distance by population affected estimated prevalence rates by contrasting the fit of competing models that differed in which covariates were included in the model structure. Mean daily temperature and wind speed were estimated for habitats used during summer for each swan population using weather data [24] for the breeding season (June through August) from 2000 through 2010 from the reporting station nearest to PTT deployment/blood sample collection locations (Table 1, Figure 1). Duration of autumn and spring migration was defined as the number of days between the time a bird initiated migration (date of first location away from a breeding area or wintering area) and the time it arrived (was first detected) at a wintering site or breeding area. There was rarely substantial distal (>50 km) movement prior to actual departure for migration, so timing of initiation of migration was rarely equivocal. The distance birds moved during migration was calculated by summing the total distances moved between sequential locations during that entire migratory period, including local movement at staging areas [22]. Therefore, mean annual migration distance was calculated by summing the mean annual distance of autumn and spring migrations by population.

**Table 1:** Weather data during the breeding season (June through August) from 2000 through 2010 for the closest weather station to locations in Alaska where swans from three populations were instrumented with transmitters and blood samples were collected.

	Arctic Coastal Plain	Bristol Bay Lowland	Lower Alaska Peninsula
weather station	Deadhorse	King Salmon	Cold Bay
mean daily temperature (°C)	5.9 (5.6–6.1)	12.1 (11.9–12.2)	9.6 (9.5–9.7)
mean daily wind speed (km/hr)	18.8 (18.3–19.3)	13.0 (12.7–13.4)	22.7 (22.1–23.3)

The 95% confidence interval for each mean daily value is reported in parentheses.

We used an information-theoretic approach to distinguish among competing models of estimated prevalence rate, wherein those with the lowest Akaike Information Criterion (AIC) score were the most likely within our model set [25]. Models were constructed and evaluated in program MARK [26]. The population affiliation covariate used in models subsumed both environmental and migratory variables that could explain differences in hematozoa infection rates. Therefore we used the coefficient of determination (R^2^) to assess the amount of variation in infection rates among populations that could be explained by temperature, wind speed, migration duration, and migration distance. More explicitly, these R^2^ values were calculated using deviance values [27], which represent fit of the model to the data, as follows: (deviance[model with no differences among populations] - deviance[model incorporating an ecological covariate, such as migration distance or wind speed])/(deviance[model with no population differences in prevalence] - deviance[model wherein all 3 populations differ in prevalence]). For some samples, sequencing efforts resulted in poor quality, single stranded, and/or short fragments (≤380 bp) of parasite mtDNA. Results for these samples were considered to be unresolved, as products could have resulted from poor amplification due to co-infection [20], degraded or low concentrations of template parasite mtDNA, secondary structures in template mtDNA, or as the result of laboratory techniques (e.g., reading errors encountered during automated sequencing or undetected contamination). Therefore, to assess the sensitivity of our results to the presence of these unresolved samples, we conducted two sets of analyses – one where we treated unresolved samples as detections of hematozoa (positives) and another where we treated unresolved samples as cases where blood parasites were not detected (negatives).

Additionally, we conducted separate analyses of *Leucocytozoon* prevalence and *Haemoproteus/Parahaemoproteus/Plasmodium* prevalence. New models that account for detection rates (i.e., occupancy models) have been developed to explicitly evaluate whether prevalence rates in two species, or species groups, are related at the individual level [28]. However, these models could not be successfully applied here as our data set is not large enough to support the significant increase in numbers of parameters that would need to be estimated. If we ignored detection rates (i.e., assumed they are perfect), then we could evaluate whether the frequency of joint occurrence of *Leucocytozoon* and *Haemoproteus/Parahaemoproteus/Plasmodium* in individuals is greater than expected, given the individual occurrence of these taxonomic groups. Variances and confidence intervals (c.i.) for these occurrence rates were computed based on a binomial distribution [29].

### Phylogenetic Assignment of Hematozoa

Phylogenetic analyses were used to taxonomically assign sequence data for hematozoa mtDNA to three broad taxonomic groups: (1) *Leucocytozoon* (2) *Haemoproteus*/*Parahaemoproteus* and (3) *Plasmodium*. Only sequences resulting from positive samples as previously defined and containing two or fewer ambiguous bases were included in phylogenetic analyses. Samples with two or fewer ambiguous bases could either be simultaneously infected with closely related parasites or may be the product of variation within a single parasite mtDNA genome [30]. Sequences containing three or more ambiguous nucleotide sites were presumed to be probable co-infections and therefore excluded from phylogenetic assignment. Phylogenies were constructed by comparing hematozoa mtDNA cyt *b* sequence data to reference sample sequences (Table S1) obtained from NCBI. Ten reference mtDNA cyt *b* sequences from each of four genera of hematozoa originating from samples of wild migratory birds were selected to represent *Leucocytozoon*, *Haemoproteus*, *Parahaemoproteus*, and *Plasmodium* lineages. Sequences were aligned using Sequencher version 4.7 and edited to a final contig of 423 nucleotides.

Phylogenies were created using MEGA version 4.0.2 [31] and MrBayes version 3.1.2 [32]. The maximum composite likelihood model for nucleotide sequences was used in MEGA with 10,000 bootstrap replicates to generate neighbor-joining trees. In MrBayes, each analysis was run for at least 1 · 10^6^ generations (or until the standard deviation of split frequencies was <0.01) using four heated chains following a burn-in of 5000 generations. Average posterior probabilities of the 50% majority rule consensus tree topologies were estimated using a sampling of likelihood parameters every 100 generations. Trees were visualized using FigTree [33].

### Assessing Haplotype Diversity

Haplotype diversity of hematozoa mtDNA cyt *b* sequences was assessed for Alaska tundra swan populations by summarizing the frequency of unique haplotypes and creating a median-joining minimum spanning network [34] using Network version 4.6 (available at www.fluxus-engineering.com). Only those haplotypes without ambiguous nucleotide positions were used to generate the network of observed mtDNA diversity. Parasite haplotypes detected in tundra swans sampled in Alaska were compared to 990 lineages available on the MalAvi public database [35] as of 9 July 2012 to identify sequences identical to those previously reported for hematozoa infecting wild birds.

## Results

### Migratory Patterns of Alaska Tundra Swans

**Figure 2:**
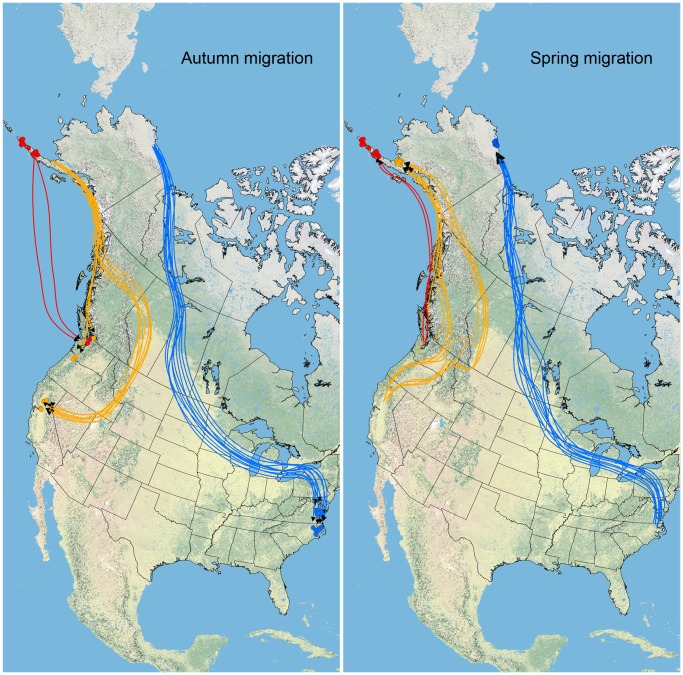
Autumn and spring migratory pathways for Arctic Coastal Plain (blue), Bristol Bay Lowland (orange), and Lower Alaska Peninsula (red) populations of Alaska tundra swans from 2008 through 2011 based on locations of birds marked with satellite transmitters. Locations representing the endpoint of migratory movements are indicated by circles.

PTT marked swans from each of three sample populations followed migration patterns between summer and wintering areas as previously documented [14], [17], [35]. Generally, Arctic Coastal Plain tundra swans departed molting areas in autumn and migrated southeast across the Northwest Territories and Prairie Pothole regions of Canada, continued east-southeast across the eastern Great Plains and Great Lakes regions of the United States, and arrived at wintering areas at Chesapeake Bay and eastern North Carolina (Figure 2). Spring migratory movements generally followed the same pathway taken southward the previous autumn (Figure 2). Tundra swans from the Arctic Coastal Plain migrated a mean annual distance of 11,932 km and spent a mean of 153.0 days in transit.

Tundra swans marked at the Bristol Bay Lowlands generally migrated southeast in autumn along the coast of Alaska before either continuing along the west coast of North America to the Pacific Northwest, or migrating inland across British Columbia, down the east side of the northern Rocky Mountains, to wintering areas in the Pacific Northwest and northern California (Figure 2). In spring movements were generally north, along coastal or interior British Columbia or northeast to western Alberta and then northwest to southern Alaska (Figure 2). Swans then generally flew west along the southern Alaska coast to summer sites at the Bristol Bay Lowlands (Figure 2). Birds from this population migrated a mean annual distance of 7347 km and spent a mean of 101.2 days in transit.

Tundra swans from the Lower Alaska Peninsula generally moved between locations along the peninsula throughout autumn and winter months and made few long distance movements with the exception of two individuals that migrated across the Gulf of Alaska to Puget Sound during the autumns of 2008 and 2010, returning via a similar, but more near-shore, route in the spring of 2009 and 2011, respectively (Figure 2). Tundra swans from the Lower Alaska Peninsula migrated a mean distance of 393 km throughout autumn and spring months and spent a mean of 1.2 days in transit as calculated using data for all birds for which transmitters were active throughout the migration period. However, the mean annual migration distance was 5503 km and mean annual time in transit was 17.0 days when considering only the two swans from the Lower Alaska Peninsula that made movements away from breeding and molting areas.

### Hematozoa Detection

A 470 bp fragment from the mtDNA cytochrome oxidase I gene was successfully amplified from all but a single sample from the Bristol Bay Lowland tundra swan population confirming the competency of 307 of 308 DNA extractions. Results for the one sample that failed to yield a PCR product at the mtDNA cytochrome oxidase I gene were therefore dropped from subsequent analyses. Of the remaining 307 samples, 135 tested positive on one or more attempts to detect hematozoa infection (44.0%). A total of 108 samples tested positive for *Leucocytozoon* parasites (35.2%; Table S2) and 71 samples tested positive for *Haemoproteus*/*Parahaemoproteus/Plasmodium* infection (23.1%; Table S2). If infection with *Leucocytozoon* parasites is independent of infection by *Haemoproteus/Parahaemoproteus/Plasmodium* species, then we would expect approximately 8.1% to be positive for infection for both nested PCR detection tests (the product of 35.2% and 23.1%). The observed rate of co-infection (14.3%, 95% c.i. 10.4% –18.2%, n = 44 samples) was slightly higher than expected. The proportion of samples from which *Leucocytozoon* infection was detected among the Arctic Coastal Plain, Bristol Bay Lowland, and Lower Alaska Peninsula populations of Alaska tundra swans was 0.33, 0.68, and 0.05, respectively as determined by nested PCR (Table S2). The proportion of samples from which *Haemoproteus/Parahaemoproteus/Plasmodium* parasites were detected was 0.03, 0.62, and 0.04, respectively from the same populations (Table S2).

### Estimating Prevalence Rates and Effects of Covariates

Estimates of hematozoa prevalence and effects of covariates were assessed for three populations of tundra swans under two conditions: (1) treating unresolved samples as either positive or (2) negative for infection. Estimates for prevalence rates, detection probabilities, and model selection criteria varied slightly between these two sets of analyses; however, statistical relationships and model support were unchanged. Therefore, we present only results of analyses where unresolved samples were treated as negative for hematozoa infection unless identified otherwise. Estimates of hematozoa prevalence rates, detection probabilities, and model selection criteria for analyses treating unresolved samples as positive for infection have been included as supplemental material (Tables S3, S4, Figure S1).

Estimated prevalence rates of blood parasites differed among populations, as based on the model with the lowest AIC value (Table 2). Statistical evaluation of the simultaneous influence of multiple covariates on prevalence rates among populations was precluded by too few degrees of freedom in our analyses. Therefore R^2^ values were used to gain insight as to whether the four ecological covariates we considered may contribute to population differences in hematozoa prevalence. Mean daily wind speed had high R^2^ values for both *Leucocytozoon* and *Haemoproteus*/*Parahaemoproteus/Plasmodium* analyses, suggesting some influence of wind or a correlated factor on parasite prevalence. Temperature also had a high R^2^ value, but only in relation to *Haemoproteus*/*Parahaemoproteus/Plasmodium* prevalence (Table 2).

**Table 2:** Model selection results using the information theoretic approach^1^.

Predicting *Leucocytozoon* infection							
**Model**	**AICc**	**ΔAICc**	**AICc Weights**	**Model Likelihood**	**Parameters**	**Deviance**	**R-squared**
{ψ area, ρ hematozoa prevalence}	436.4206	0	0.82122	1	4	428.2882	
{ψ wind, ρ hematozoa prevalence}	439.4699	3.0493	0.17878	0.2177	3	433.3907	0.949981718
{ψ migration duration, ρ hematozoa prevalence}	500.5873	64.1667	0	0	3	494.5081	0.350866118
{ψ migration distance, ρ hematozoa prevalence}	504.4218	68.0012	0	0	3	498.3426	0.313277661
{ψ temperature, ρ hematozoa prevalence}	516.8494	80.4288	0	0	3	510.7702	0.191453613
{ψ, ρ hematozoa prevalence}	534.3404	97.9198	0	0	2	530.3009	
**Predicting ** ***Haemoproteus*** **/** ***Parahaemoproteus*** **/** ***Plasmodium*** ** infection**							
**Model**	**AICc**	**ΔAICc**	**AICc Weights**	**Model Likelihood**	**Parameters**	**Deviance**	**R-squared**
{ψ area, ρ hematozoa prevalence}	277.4747	0	0.95578	1	4	269.3423	
{ψ wind, ρ hematozoa prevalence}	283.6705	6.1958	0.04315	0.0451	3	277.5913	0.938686787
{ψ temperature, ρ hematozoa prevalence}	291.0593	13.5846	0.00107	0.0011	3	284.9801	0.883767273
{ψ migration duration, ρ hematozoa prevalence}	405.5536	128.0789	0	0	3	399.4744	0.032753401
{ψ migration distance, ρ hematozoa prevalence}	407.21	129.7353	0	0	3	401.1308	0.020441702
{ψ, ρ hematozoa prevalence}	407.9204	130.4457	0	0	2	403.881	

1 Unresolved samples considered as negative for infection.

**Figure 3:**
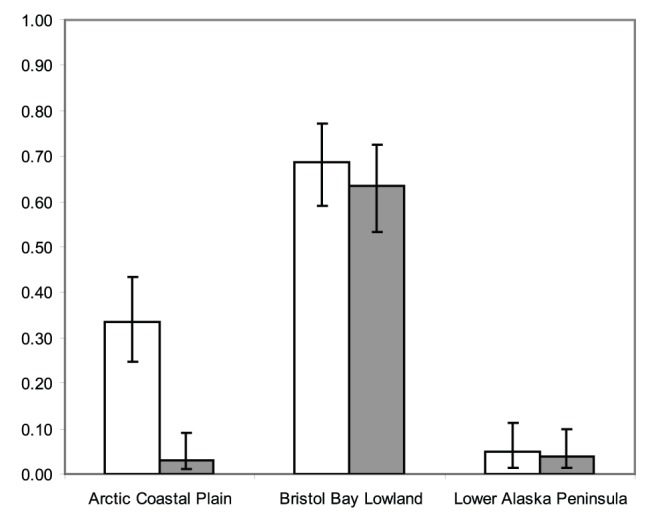
Estimated prevalence of *Leucocytozoon* (white bars) and *Haemoproteus*/*Parahaemoproteus*/*Plasmodium* (gray bars) infection in populations of Alaska tundra swans with unresolved samples considered as negative for infection. Associated 95% confidence intervals for estimates are indicated with error bars.

The detection probabilities for *Leucocytozoon* and *Haemoproteus*/*Parahaemoproteus/Plasmodium* infection were relatively high (≥0.86) based on the top model in each analysis (Table 3). *Leucocytozoon* infection was significantly different across all populations of Alaska tundra swans examined with the highest estimated prevalence occurring in Bristol Bay Lowland birds (0.69, 95% c.i. 0.59–0.77) followed by Arctic Coastal Plain (0.33, 95% c.i. 0.25–0.43) and Lower Alaska Peninsula swans (0.05, 95% c.i. 0.02–0.11; Figure 3). Similarly, *Haemoproteus*/*Parahaemoproteus/Plasmodium* infection was estimated to be highest in the Bristol Bay Lowland tundra swan population (0.63, 95% c.i. 0.53–0.72; Figure 3). Lower, statistically equivalent prevalence of *Haemoproteus*/*Parahaemoproteus/Plasmodium* infection was estimated for the Arctic Coastal Plain (0.03, 95% c.i. 0.01–0.09) and Lower Alaska Peninsula (0.04, 95% c.i. 0.01–0.10) populations (Figure 3).

**Table 3:** Estimated detection probabilities for *Leucocytozoon* and *Haemoproteus*/*Parahaemoproteus*/*Plasmodium* infections in Alaska tundra swans from the highest ranking model using AIC^1^.

	detection probability	standard error	lower 95% confidence interval	upper 95% confidence interval
*Leucocytozoon*	0.8833476	0.0252980	0.8239483	0.9245412
*Haemoproteus*/*Parahaemoproteus*/*Plasmodium*	0.8596952	0.0414497	0.7575373	0.9231755

1 Unresolved samples considered as negative for infection.

### Phylogenetic Assignment of Hematozoa

**Figure 4:**
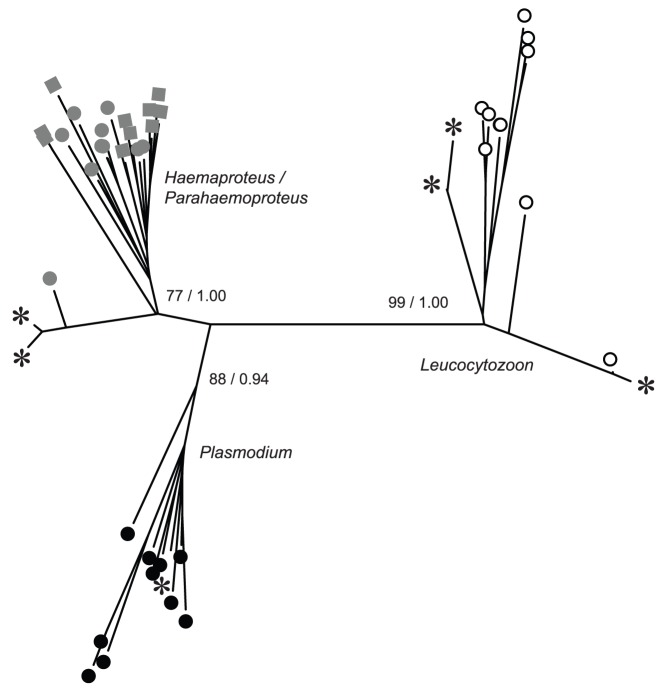
Phylogenetic assignment of hematozoa mitochondrial DNA cytochrome *b* sequences originating from Alaska tundra swans (asterisks). Closely related haplotypes are represented by a single asterisk. Reference sequences for *Leucocytozoon* (white circles), *Plasmodium* (black circles), *Haemoproteus* (grey circles), and *Parahaemoproteus* (grey squares) parasites were obtained from the National Center for Biotechnology Information. Bootstrap support values and posterior probabilities for differentiation of broad taxonomic groups are indicated.

Phylogenetic assignment was successfully used to differentiate 156 double-stranded hematozoa mtDNA sequences into three broad taxonomic groups: (1) *Leucocytozoon* spp. (2) *Haemoproteus*/*Parahaemoproteus* spp. and (3) *Plasmodium* spp. as confirmed by relatively high bootstrap support (≥77%) and posterior probability (≥0.94) values (Figure 4). Sequence totals of 100, 55, and 1 were phylogenetically assigned as being *Leucocytozoon* spp., *Haemoproteus*/*Parahaemoproteus* spp., and *Plasmodium* spp., respectively. The proportion of tundra swans samples from which *Leucocytozoon* spp. were detected from the Bristol Bay Lowland population was 0.61 as compared to 0.32 for the Arctic Coastal Plain population and 0.05 for the Lower Alaska Peninsula population based on phylogenetic assignment of haplotypes (Table 4). Similarly, the proportion of swan samples from the Bristol Bay population from which *Haemoproteus*/*Parahaemoproteus* spp. was detected was 0.47 as compared to 0.03 and 0.04 from the Arctic Coastal Plain and Lower Alaska Peninsula populations, respectively (Table 4). *Plasmodium* parasites were only detected in a single sample from the Bristol Bay Lowland population (Table 4).

**Table 4:** Number and proportion of samples from three populations of Alaska tundra swans infected by *Leucocytozoon*, *Haemoproteus*/*Parahaemoproteus*, *and Plasmodium* parasites as assigned using phylogenetic analyses.

	Arctic Coastal Plain	Bristol Bay Lowland	Lower Alaska Peninsula
number of samples examined	100	103	104
*Leucocytozoon* positive	32 (0.32)	63 (0.61)	5 (0.05)
*Haemoproteus*/*Parahaemoproteus* positive	3 (0.03)	48 (0.47)	4 (0.04)
*Plasmodium* positive	0 (0.00)	1 (0.01)	0 (0.00)

Reported values do not include presumed co-infections or unresolved samples (see Materials and methods).

### Haplotype Diversity

**Figure 5:**
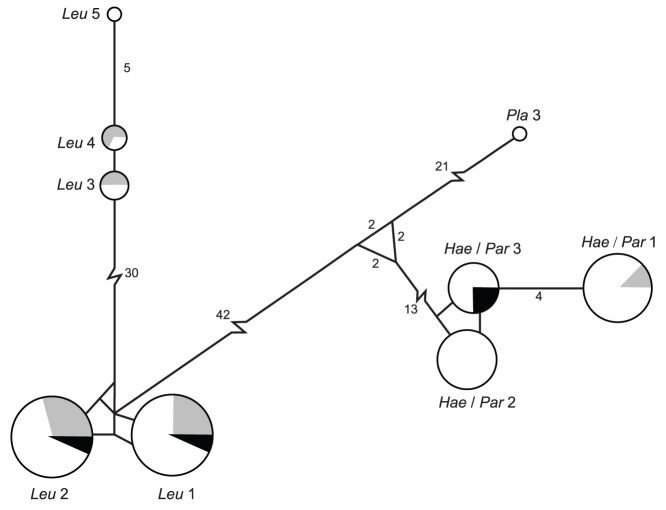
Minimum spanning network for hematozoa mitochondrial DNA cytochrome *b* haplotypes detected in Alaska tundra swans. Circles are drawn proportional to the frequency at which haplotypes were observed. Shading represents the population from which haplotypes originated: gray (Arctic Coastal Plain), white (Bristol Bay Lowland), and black (Lower Alaska Peninsula). A single mutation separates nodes unless explicitly indicated by number. Lines separating nodes are drawn to scale unless indicated by a break. Parasite taxa have been abbreviated in haplotype names (Leu = *Leucocytozoon*, Hae/Par = *Haemoproteus*/*Parahaemoproteus* and Pla = *Plasmodium*).

A total of nine unique haplotypes were observed among the 124 mtDNA cyt *b* hematozoa sequences analyzed from all populations of tundra swans sampled in Alaska (Table 5). Genbank accession numbers for these nine haplotypes are: JQ314220– JQ314228. Haplotypes were identified as *Leucocytozoon* spp. (n = 5) *Haemoproteus*/*Parahaemoproteus* spp. (n = 3) and *Plasmodium* sp. (n = 1) as inferred from phylogenetic assignment (Table 5). The greatest number of haplotypes was detected in the Bristol Bay Lowland population (n = 9) followed by the Arctic Coastal Plain (n = 5) and the Lower Alaska Peninsula populations (n = 3; Table 5, Figure 5). All haplotypes for *Leucocytozoon*, *Haemoproteus*/*Parahaemoproteus*, and *Plasmodium* species were detected in samples from the Bristol Bay Lowland population (Table 5, Figure 5). Four *Leucocytozoon* haplotypes and a single *Haemoproteus*/*Parahaemoproteus* sequence were detected in samples from the Arctic Coastal Plain tundra swan population (Table 5, Figure 5). Two *Leucocytozoon* haploypes and a single *Haemoproteus*/*Parahaemoproteus* haplotype were detected in samples from swans sampled at the lower Alaska Peninsula (Table 5, Figure 5).

**Table 5:** The distribution of 124 hematozoa mitochondrial DNA cytochrome *b* haplotypes observed among three populations of tundra swans in Alaska.

	Arctic Coastal Plain	Bristol Bay Lowland	Lower Alaska Peninsula
	(n = 24)	(n = 93)	(n = 7)
*Leucocytozoon* haplotype 1	8	22	2
*Leucocytozoon* haplotype 2	9	20	2
*Leucocytozoon* haplotype 3	2	2	0
*Leucocytozoon* haplotype 4	2	1	0
*Leucocytozoon* haplotype 5	0	1	0
*Haemoproteus/Parahaemoproteus* haplotype 1	3	20	0
*Haemoproteus/Parahaemoproteus* haplotype 2	0	17	0
*Haemoproteus/Parahaemoproteus* haplotype 3	0	9	3
*Plasmodium* haplotype 1	0	1	0

Three haplotypes detected in tundra swans sampled in Alaska were identical to lineages reported on the MalAvi database. *Haemoproteus*/*Parahaemoproteus* mtDNA cytochrome *b* haplotype 1 was identical to lineage ANACRE01 previously identified from a sample collected from a green-winged teal (*Anas crecca*). *Haemoproteus*/*Parahaemoproteus* mtDNA cytochrome *b* haplotype 2 was identical to lineage CYGNUS01 which has been detected in samples originating from a tundra swan and a mallard (*Anas platyrhynchos*) sampled in Minnesota. *Plasmodium* mtDNA cytochrome *b* haplotype 1 was identical lineage BT7 which has been identified in samples collected from numerous avian taxa throughout the Holarctic including a pectoral sandpiper (*Caldris melanotos*) sampled in Alaska.

## Discussion

We observed differences in seasonal distribution and migration patterns between tundra swans marked in Alaska along the Arctic Coastal Plain, the Bristol Bay Lowlands, and the Lower Alaska Peninsula (Figure 2), corroborating earlier designation of discrete populations [36]. We also detected differences in the prevalence and genetic diversity of blood parasites among these populations (Table 5, Figures 3 and 5). Although no single environmental covariate could explain population differences in hematozoa prevalence, ecological characteristics of summer use areas (wind and temperature) were the best fitting of those considered and consistent with predictions of how habitats may influence patterns of hematozoa infection in wild bird populations [1]–[2]. Our results do not support migration distance or duration as a predictive covariate of hematozoa infection in tundra swans [9].

**Figure 6:**
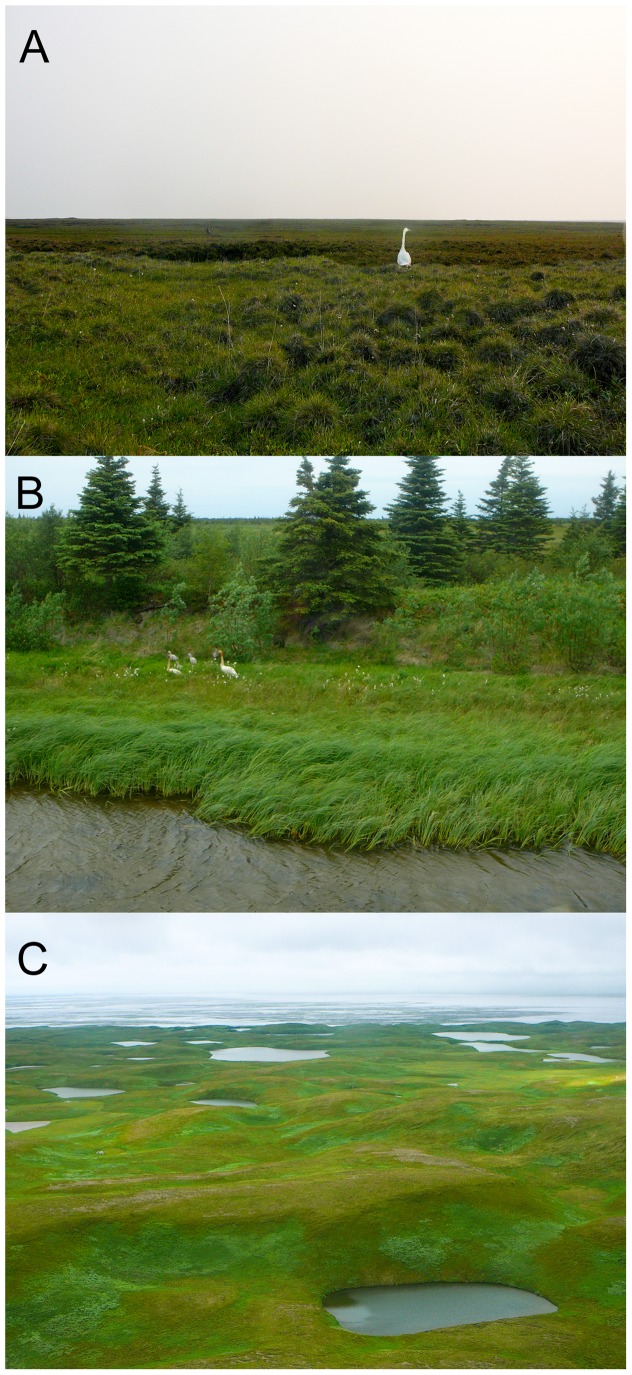
Summer habitats used by tundra swans in Alaska. (A) the Arctic Coastal Plain, (B) the Bristol Bay Lowlands, and (C) the Lower Alaska Peninsula.

In this study, the Bristol Bay Lowland tundra swan population was found to have the highest prevalence and diversity of hematozoa (Table 5, Figures 3, 5, and S5), most likely a function of ecological characteristics of summer habitats and exposure of swans to insect vectors. The Bristol Bay Lowlands are located at the limit of the North American boreal forest and are characterized by discontinuous woodlands adjacent to wet meadow habitats (Figure 6) [37]. Forested habitats in this region likely support ornithophilic mosquitoes, blackflies, and biting midges capable of accessing nearby swans during active feeding flights for blood meals. Furthermore, daily mean wind speeds are lower at this location than at other areas investigated in this study (Table 1) which may facilitate flight of biting insect vectors. Additionally, mean summer temperatures, which may limit some stages of hematozoa development [8], are higher at this site than at other sampling locations in Alaska examined in this study (Table 1). Therefore, hematozoa may have a longer period during which to develop at the Bristol Bay Lowlands than at other locations from which swans were sampled in this study. The relatively high prevalence rate of hematozoa in tundra swans at this location (Figure 3) is consistent with rates of hematozoa detection (≥57%) in avian taxa sampled near the boreal forest edge in Canada near Ungava Bay, Quebec [38] and Churchill, Manitoba [2].

Although our data do not support migration distance or duration as being predictive of hematozoa infection, tundra swans from the Bristol Bay Lowland population did undertake long annual migrations between the northeastern Alaska Peninsula and wintering areas in British Columbia, Washington, Oregon, and California (Figure 2). The acquisition of hematozoa infections has been demonstrated at wintering areas of migratory birds including California [39]. Furthermore, stop-over and staging areas used by swans throughout migration are additional areas of potential parasite exposure, including locations in Alberta where active transmission of *Leucocytozoon*, *Haemoproteus/Parahaemoproteus*, and *Plasmodium* parasites has been demonstrated in trumpeter swans (*Cygnus buccinator*) [40]. Therefore, although our data suggest that ecological conditions at the Bristol Bay Lowlands may explain the prevalence of hematozoa in this population, swans could be exposed to blood parasites at different points in time and space throughout the year.

The prevalence and diversity of hematozoa detected in the Arctic Coastal Plain population of tundra swans was found to be intermediary for *Lecocytozoon* spp. and relatively low for *Haemoproteus*/*Parahaemoproteus* spp. (Table 5, Figures 3, 5, and S5). Infection of tundra swans by *Plasmodium* spp. was not detected in this population (Table 4). Nesting habitats used by the Arctic Coastal Plain population during summer are well beyond the northern extent of the North American boreal forest, providing little or no tall vegetative cover for biting insects (Figure 6) [41]–[42]. Furthermore, the mean daily wind speed at the Arctic Coastal Plain throughout summer months was higher than at the Bristol Bay Lowlands (Table 1) and appreciably greater than necessary to inhibit active flight of biting insects [43]–[44]. These ecological variables may reduce the exposure of swans to suitable vectors of hematozoa. Furthermore, low mean summer temperatures along the Arctic Coastal Plain (Table 1) may inhibit the development of certain life stages of hematozoa, particularly *Plasmodium* and *Haemoproteus*/*Parahaemoproteus* parasites [7]–[8]. Indeed, previous studies found no evidence for active transmission of hematozoa beyond the Arctic Circle in resident Nearctic avian species [2], [37]. However, active transmission of *Leucocytozoon simondi* has been demonstrated beyond the Arctic Circle in the Palearctic [8], likely as the result of sporogony adapting to develop at low temperatures in high latitudes [45]. Similar adaptation of Nearctic *Leucocytozoon* spp. could explain the intermediary prevalence of these parasites in the Arctic Coastal Plain population of swans.

The long annual migration distance of tundra swans marked at the Arctic Coastal Plain (Figure 2) could also contribute towards the intermediary prevalence of *Leucocytozoon* infections in this population, even if unsupported as a predictive covariate. Low rates of parasite transmission at breeding and molting areas due to environmental conditions could be supplemented by exposure to parasites during migration and at wintering areas. Swans from the Arctic Coastal Plain population traversed large expanses of the northern boreal forest during both autumn and spring migrations in each year and passed through the ranges of numerous species of blackflies, biting midges, and mosquitoes known to be the vectors of *Leucocytozoon*, *Haemoproteus*/*Parahaemoproteus*, and *Plasmodium* parasites, respectively [1], [39], [46]. However, it remains unclear if insect vectors were active and carrying infectious life stages of hematozoa during times when tundra swans were using stop-over and staging areas throughout migration. Seasonal transmission of *Haemoproteus columbae* by hippoboscid flies was found to be highest among feral pigeons (*Columba livia*) in autumn and winter in Michigan [47]; and, *Leucocytozoon smithi* was actively transmitted among domestic turkeys (*Meleagris gallopavo*) by simuliid flies in South Carolina throughout the year [48]. This suggests that vectors may spread hematozoa among birds at mid-latitude locations of North America temporally and spatially proximate to those used by Arctic Coastal Plain tundra swans outside of the summer breeding season.

The Lower Alaska Peninsula tundra swan population had the lowest prevalence and diversity of avian hematozoa (Table 5, Figures 3, 5, and S5). Habitats along the Lower Alaska Peninsula are characterized by large expanses of lowland coastal tundra with relatively little or no tree cover (Figure 6), low mean monthly temperatures throughout the year, and persistent high winds (Table 1) [17]. The low temperatures and high winds that occur at this location throughout the breeding season (Table 1) could both limit the exposure of breeding tundra swans to biting insect vectors and inhibit the development of blood parasites. Similar to our findings at the Lower Alaska Peninsula, a low incidence (2.9%) of hematozoa infection has been documented in tundra nesting geese in sub-arctic Canada near Hudson Bay [49].

Lower Alaska Peninsula tundra swans made infrequent migratory movements in autumn to mid-latitude wintering locations (Figure 2), which likely minimized exposure to vectors outside of the breeding season and may have further contributed to the low rates of infection detected in this study. However, it remains unclear if the low prevalence of blood parasites estimated for this population (Figure 3) is representative of infrequent transmission of hematozoa at the Lower Alaska Peninsula during summer or the result of migratory movements of a proportion of individuals from this population to mid-latitude wintering areas where infections are acquired.

In this study, we explored patterns of hematozoa infection in discrete populations of tundra swans that breed in Alaska using empirical data for host migration, ecological conditions at breeding areas, and parasite prevalence/genetics while controlling for potential host species effects. However, we recognize that several important assumptions were made which should be considered when interpreting our results. A central assumption of this study is that migratory movements of instrumented birds are representative of those which were screened for hematzooa infection and *vice versa*. Similarly, weather data for microhabitats used by individual swans were not obtained and therefore mean daily values for the nearest reporting station throughout the annual breeding season were assumed to be representative of conditions encountered by birds during summer. As migration parameters and ecological conditions at breeding areas were calculated with one sample population per region, the power of our current model selection approach is limited. Future sampling across broader areas of each habitat region will allow better assessments of within as well as among region variation in environmental factors that may contribute to hemosporidian infection rates. Additionally, it was assumed that ecological conditions at breeding areas in Alaska were important in explaining patterns of hematozoa infection in tundra swans. However, as infections by *Leucocytozoon*, *Haemoproteus*/*Parahaemoproteus*, and *Plasmodium* parasites have all been demonstrated to persist across years with annual relapses most commonly occurring during the breeding season [8], it cannot be conclusively determined if initial infections occurred at or away from breeding areas. This consideration is particularly relevant to this study as tundra swans are long-lived [50] and most blood samples were collected from after-second-year birds. Finally, as all blood samples analyzed in this study were collected during the breeding season in Alaska when temperatures are generally highest (July and August), it was assumed that our sampling approach minimized temporal variation in levels of infection among populations.

Future examination of hematozoa in Alaska breeding tundra swans could build upon results of this study by sampling cygnets, making peripheral blood smears, sampling across broader regions within each habitat type, and conducting vector screening. Samples collected from hatch-year birds at or near breeding grounds would more clearly demonstrate transmission at summer habitats. Peripheral blood smears could be used to better assess co-infection rates, to identify parasite morpho-species, and to assess levels of parasitemia. Vector screening to assess the relative abundance of biting insects and to determine if they are carrying infectious life stages of hemosporidians could be useful for identifying whether blood parasite transmission is limited by the absence of suitable vectors or the effects of ecological variables such as temperature and wind. Additionally, a comparative study of hematozoa infection in tundra swans and trumpeter swans sampled in Alaska would be of interest, as trumpeter swans nest almost exclusively in boreal forest habitats and migrate shorter distances compared to tundra swans. The addition of these components to future studies would provide further insight into patterns of hematozoa infection in migratory birds breeding in Alaska and provide additional baseline data to assess potential changes through time.

## Supporting Information

Figure S1 **Estimated prevalence of** ***Leucocytozoon*** **(white bars) and** ***Haemoproteus*** **/** ***Parahaemoproteus*** **/** ***Plasmodium*** **(gray bars) infection in populations of Alaska tundra swans with unresolved samples considered as positive for infection.** Associated 95% confidence intervals for estimates are indicated with error bars.
(XLSX)

Table S1 **Reference sequences used to create hematozoa phylogenies.**
(XLSX)

Table S2 **The number and proportion (in parentheses) of samples testing positive for** ***Leucocytozoon*** **and** ***Haemoproteus*** **/** ***Parahaemoproteus*** **/** ***Plasmodium*** **infection from populations of Alaska tundra swans as determined by nested PCR.** The number and proportion of unresolved samples is also reported.
(XLSX)

Table S3 **Model selection results using the information theoretic approach with unresolved samples considered as positive for infection.**
(XLSX)

Table S4 **Estimated detection probabilities for** ***Leucocytozoon*** **and** ***Haemoproteus*** **/** ***Parahaemoproteus*** **/** ***Plasmodium*** **infections in Alaska tundra swans from the highest ranking model using AIC where unresolved samples were considered positive for infection.**
(XLSX)
